# Effects of the Molecular Weight and the Degree of Deacetylation of Chitosan Oligosaccharides on Antitumor Activity

**DOI:** 10.3390/ijms12010266

**Published:** 2011-01-06

**Authors:** Jae Kweon Park, Mi Ja Chung, Ha Na Choi, Yong Il Park

**Affiliations:** Department of Biotechnology, The Catholic University of Korea, Bucheon, Gyeonggi-do 420-743, Korea; E-Mails: jamyeong@yahoo.co.kr (J.K.P.); mimichung@hanmail.net (M.J.C.); glitter_hana@naver.com (H.N.C.)

**Keywords:** chitosan oligosaccharides, antitumor activity, MALDI-TOF MS, molecular weight, degree of deacetylation

## Abstract

Effects of the degree of deacetylation (DDA) and the molecular mass of chitosan oligosaccharides (CTS-OS), obtained from the enzymatic hydrolysis of high molecular weight chitosan (HMWC), on antitumor activity was explored. The DDA and molecular weights of CTS-OS were determined by matrix-assisted laser desorption/ionization-mass spectrometry (MALDI-TOF MS) analysis. The CTS-OS were found to be a mixture of mainly dimers (18.8%), trimers (24.8%), tetramers (24.9%), pentamers (17.7%), hexamers (7.1%), heptamers (3.3%), and octamers (3.4%). The CTS-OS were further fractionated by gel-filtration chromatography into two major fractions: (1) COS, consisting of glucosamine (GlcN)_n_, n = 3–5 with DDA 100%; and (2) HOS, consisting of (GlcN)_5_ as the minimum residues and varying number of *N*-acetylglucosamine (GlcNAc)_n_, n = 1–2 with DDA about 87.5% in random order. The cytotoxicities, expressed as the concentration needed for 50% cell death (CC_50_), of CTS-OS, COS, and HOS against PC3 (prostate cancer cell), A549 (lung cancer cell), and HepG2 (hepatoma cell), were determined to be 25 μg·mL^−1^, 25 μg·mL^−1^, and 50 μg·mL^−1^, respectively. The HMWC was approximately 50% less effective than both CTS-OS and COS. These results demonstrate that the molecular weight and DDA of chitosan oligosaccharides are important factors for suppressing cancer cell growth.

## 1. Introduction

Chitin is the second most abundant naturally occurred homopolysaccharide composed of β-(1,4)-linked-d-*N*-acetylglucosamine (GlcNAc) after cellulose. Chitosan is a linear heteropolysaccharide composed of β-(1,4)-linked-d-glucosamine (GlcN) and *N*-acetylglucosamine (GlcNAc), which is derived from chitin. For several decades, the study on chitosan has attracted interest in converting it into more soluble form of chitosan-oligosaccharides, hereafter referred to CTS-OS, which exhibit remarkable biological activities [1–5]; they are non-toxic, biocompatible and biodegradable [6], including antibacterial antifungal, antitumor and stimulating immunoenhancing properties. It is implicated that the biological activities of CTS-OS significantly depend on their molecular weight and the degree of deacetylation (DDA) of the parental material chitosan. This is affected by the distribution pattern of GlcN/GlcNAc along the oligomeric chain of CTS-OS [7–9]. In order to study the relationship between structure and biological activity, structurally well-defined CTS-OS represent important factors to provide information regarding the development of enzymatic process with different enzymes that have different substrate specificity or cleavage patterns.

There have been numerous reports studying the antitumor activity of chitosan and its derivatives which are chemically modified [10–14]. Among them, it is known that the antitumor mechanism of chitosan nanoparticles is related to its membrane-disrupting and apoptosis-inducing activities [15,16]. However antitumor mechanism of CTS-OS with different molecular weights and DDA are not well studied yet. Although it was suggested that lower molecular weight chitosan or water-soluble chitosan may have antitumor activities in clinical use, such effects of them—including of CTS-OS having different molecular weights and DDA—are as yet unproven. Therefore, structurally well-defined CTS-OS would represent important tools for investigating the antitumor effect of CTS-OS.

In this study, we have attempted to develop a simple strategy for the preparation of more soluble forms of chitosan-oligosaccharides (CTS-OS) from the high-molecular weight chitosan (HMWC) and have investigated their structure and biological activities, especially on several tumor cell lines. For this purpose, CTS-OS were prepared by enzymatic digestion of HMWC and the relationship between their structures (molecular weights and DDA) and antitumor activities was examined against human PC3 (prostate cancer cell), human A549 (carcinomic human alveolar basal epithelial cell), and human HepG2 (hepatomacellular carcinoma) cells.

## 2. Results and Discussion

### 2.1. Gel-Filtration Chromatography of Chitosan Oligosaccharides

The chitosan-oligosaccharides (CTS-OS) in the supernatant after hydrolysis of HMWC with a chitosanase were recovered and lyophilized by the method described in the Materials and Methods to seek the feasibility of CTS-OS for the test of antitumor efficacy. CTS-OS was separated using an ultrafiltration membrane filter with a molecular weight cut off (MWCO) of 10 kDa. The major product of CTS-OS was yielded at about 95 ± 0.05% after freeze drying. The major component of CTS-OS was found to be composed of, with different levels of DDA, dimer (18.8%), trimer (24.8%), tetramer (24.9%), pentamer (17.7%), hexamer (7.1%), heptamer (3.3%), and octamer (3.4%) (Table 1).

**Figure 1:**
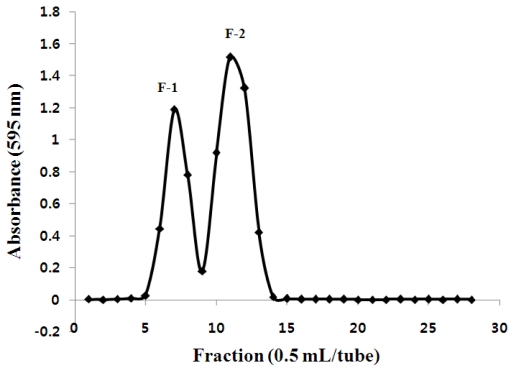
Gel-filtration column chromatography of chitosan oligosaccharides (CTS-OS). CTS-OS were fractionated through a Bio P-4 gel-filtration column at ambient temperature. F-1, fractions 5–9; F-2, fractions 10–14.

These results showed that the major component of CTS-OS derived from the enzymatic digestion of chitosan was identified to be (GlcN)_n_, n = 2–5, with yields of about 86 ± 0.2% after the freeze drying. CTS-OS was further then fractionated using a gel-filtration column chromatography (Figure 1). The carbohydrate positive fractions denoted F-1 (fractions 5–9) and F-2 (fraction 10–14) were pooled, dialyzed and freeze-dried. The two major carbohydrate-positive fractions obtained were applied to MALDI-TOF MS analysis, after which they were tentatively named chito-oligosaccharides (COS) and hetero-oligosaccharides (HOS). As shown in Table 1, DDA of CTS-OS obtained after the gel-filtration was determined to be in the range between 83.3 to 100%. Of these, COS was recovered over 86% from CTS-OS as fully deacetylated oligosaccharides by the gel-filtration column chromatography, which was shown to be a good approach for the separation of COS and HOS from CTS-OS with different DDA and molecular weights derived from the HMWC.

### 2.2. MALDI-TOF MS Analysis

**Figure 2:**
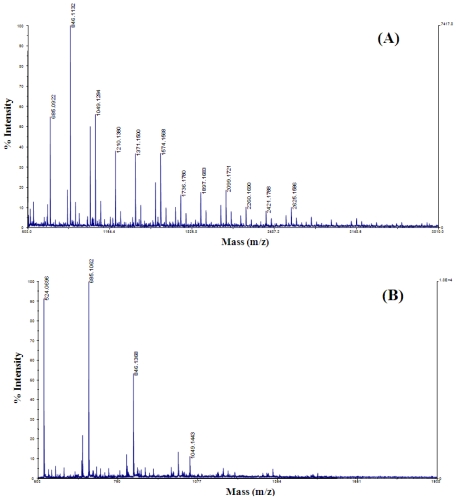
Determination of the molecular mass and the degree of deacetylation of chitosan oligosaccharides. The chitosan oligosaccharides, F-1 (**A**) and F-2 (**B**), obtained after enzymatic digestion of chitosan (HMWC) and gel-filtration chromatography were analyzed by MALDI-TOF MS spectrometry.

Since the molecular weight and the degree of *N*-deacetylation of chitosan appeared to be key factors in such biological activity, MALDI-TOF MS analysis was conducted to determine the molecular weight and primary structure of F-1 and F-2. MALDI-TOF MS analysis showed that F-1 consisted of a mixture of (HOS)_n_, n = 6–15 (Figure 2A), while F-2 consisted of a mixture of (COS)_n_, n = 3–5 (Figure 2B). F-2 contained about 67% (COS)_n_, n = 3–5 when compared to the relative percent intensity of the results obtained from the MALDI-TOF MS analysis of CTS-OS (Table 1). On the other hand, (HOS)_n_, n = 6–15 in F-1, which was detected in much lower quantity than (COS)_n_, n = 3–5 (Table 1), was obviously concentrated by Bio P-4 gel-filtration chromatography (Figure 2A). (COS)_2_ could not be separated through Bio P-4 gel filtration chromatography. The existence of different components of oligosaccharides consisting of GlcN and GlcNAc in random order in CTS-OS indicated that the parental HMWC is partially deacetylated chitosan. Nevertheless, as shown in Figure 2B, (COS)_n_, n = 3–5 in F-2 obtained from the gel filtration chromatography was the major component of the mixture of CTS-OS.

As shown in Figure 2B, (COS)_n_, n = 6 was negligible in F-2; therefore, F-2 containing (COS)_n_, n = 3–5 and F-1 containing (HOS)_n_, n = 6–15 as major component (Table 2) were used as pure chito-oligosaccharide or hetero-oligosaccharide mixtures without further separation to investigate the antitumor effect on human-derived tumor cells. Thus, the antitumor effects of HMWC, CTS-OS, (COS)_n_, n = 3–5 and (HOS)_n_, n = 6–15 on tumor cell lines were investigated to evaluate the relationship between the molecular weight and their DDA, as described in Tables 1 and 2, since no investigation was demonstrated in detail. The distribution of acetyl groups in the commercially available native chitosan is not evenly as in that produced by heterogeneous N-deacetylation of chitin. This is an interesting point for preparation of low molecular weight chitosan with different degrees of polymerization and DDA. Therefore, the preparation and characterization of the molecular weight and DDA of a series of CTS-OS precisely using MALDI-TOF MS gives rise to get better understanding of the relationship between biological functions of CTS-OS with different molecular weight and DDA.

### 2.3. Antitumor Effect of High Molecular Weight Chitosan (HMWC)

**Figure 3:**
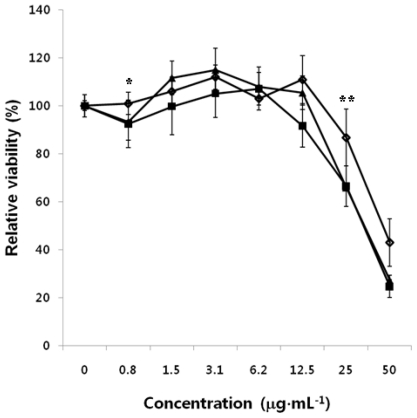
Effect of high molecular weight chitosan (HMWC) on human tumor cell lines. Cytotoxicity of HMWC against human tumor cells was performed using MTT assay *in vitro.* Human PC3 (-⋄-), human A549 (**-**▪-) and human HepG2 (**-**▪**-**) cells were exposed to the indicated amounts of chitosan for 24 h. After 24 h incubation, the absorbance of the solution was measured at 575 nm using a micro-plate reader. Data are the means ± standard deviation of three different experiments. The statistical significance of the difference between mean values was determined by the student’s t-test. *P < 0.05 and **P < 0.1 was considered significant. Experiments were performed at least in triplicates.

To examine the antitumor activity of HMWC, we carried out *in vitro* cytotoxicity experiments using MTT assay against human cells such as HepG2, A549, and PC3 as model tumor cell lines. Figure 3 shows the relative cell viability of these cells following 24 h incubation with PBS as the control or a wide range of concentrations of HMWC with from 0.75 to 50 μg·mL^−1^. While PBS alone did not show any appreciable toxicity, HMWC showed significantly higher cytotoxicity toward human HepG2 and A549 than PC3 cells, with IC_50_ values lower than 50 μg·mL^−1^. These results demonstrated that HMWC has antitumor activities against various tumor cell lines *in vitro and* is applicable as an antitumor agent based on the biodegradability and biocompatibility of HMWC, although the antitumor mechanism is not clear yet. Chitosan with different molecular weights and DDA are implicated to exhibit growth inhibitory effects against tumors in experimental animals, though the antitumor activity of chitosan seems to depend not only on molecular size but also on their chemical structure.

### 2.4. Antitumor Effect of HMWC, CTS-OS, COS and HOS on Human PC3 Cells

**Figure 4:**
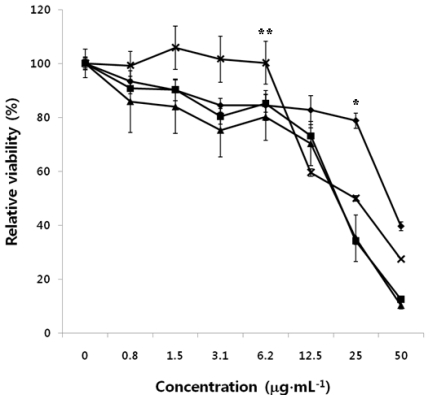
Effect of HMWC, CTS-OS, COS and HOS on human PC3 cells. Cells were exposed to the indicated amounts of HMWC (-X-), CTS-OS (**-**▪-), HOS (**-**♦**-**), and COS (**-**▴**-**) for 24 h. After 24 h incubation, cytotoxicity of CTS-OS, COS and HOS against tumor cells was determined by MTT assay. Data are mean values calculated from three independent experiments (±SD). *P < 0.05 and **P < 0.1 was considered significant.

The antitumor effects of CTS-OS, COS and HOS on human PC3 cells were evaluated *in vitro* cytotoxicity experiment with a wide range of concentrations from 0.75 to 50 μg·mL^−1^, as described above. Figure 4 shows that CTS-OS showed the strongest effect against human PC3 cells used as a model cell line, with CC_50_ values of 25 μg·mL^−1^. Treatment of COS could inhibit tumor growth similar to that of CTS-OS. In contrast, as shown in Figure 3, HOS showed slightly less growth inhibition of tumor cells with the 50% cytotoxic concentration (CC_50_) values of 50 μg·mL^−1^. Compared to HMWC, HOS showed no apparent difference in antitumor activity, though native chitosan and HOS have different extent of sizes of molecular weight. Therefore, DDA is considered as a principal features of HMWC, CTS-OS and COS. Unlike HMWC, both CTS-OS and COS attract greater interest as antitumor agents due to their water solubility.

### 2.5. Antitumor Effect of HMWC, CTS-OS, COS and HOS on Human A549 Cells

**Figure 5:**
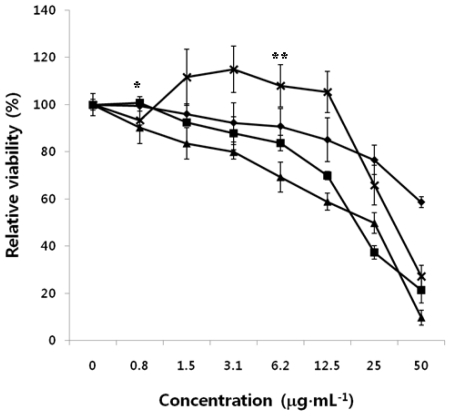
Effect of HMWC, CTS-OS, COS and HOS on human A549 cells. Cells were exposed to the indicated amounts of HMWC (-X-), CTS-OS (**-**▪-), HOS (**-**♦**-**), and COS (**-**▴**-**) for 24 h. After 24 h incubation, cytotoxicity of CTS-OS, COS and HOS against tumor cells was determined by MTT assay. Data are mean values calculated from three independent experiments (±SD). *P < 0.05 and **P < 0.1 was considered significant.

The results showed that antitumor effects of CTS-OS, COS and HOS on human A549 cells were observed from *in vitro* cytotoxicity experiment with a wide range of concentrations from 0.75 to 50 μg·mL^−1^, as described above. Figure 5 shows that CTS-OS, which consists of COS and HOS, displayed the strongest effect toward A549 cells with CC_50_ values of 25 μg·mL^−1^. Treatment of COS could inhibit tumor growth similar to that observed by CTS-OS as well. On the other hand, HOS showed much low activity against tumor cells with CC_50_ values higher than 50 μg·mL^−1^. No apparent difference of the antitumor activity of HOS compared to that of HMWC was observed.

### 2.6. Antitumor Effect of HMWC, CTS-OS, COS and HOS on Human HepG2 Cells

**Figure 6:**
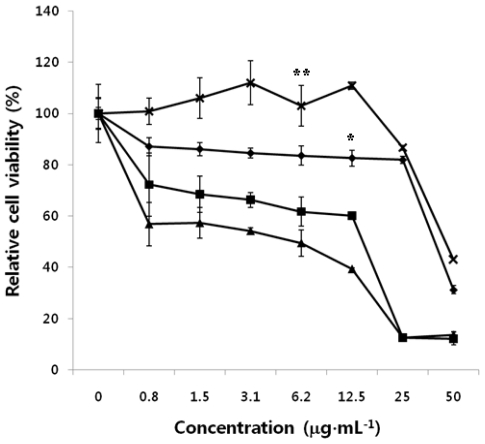
Effect of HMWC, CTS-OS, COS and HOS on human HepG2 cells. HepG2 cells were exposed to the indicated amounts of HMWC (-X-), CTS-OS (**-**▪-), HOS (**-**♦**-**), and COS (**-**▴**-**) for 24 h. After 24 h incubation, cytotoxicity of HMWC, CTS-OS, HOS and COS against tumor cells was examined by MTT assay. Data expressed are the means (±SD) of three different experiments. *P < 0.05 and * P < 0.1 was considered significant.

The antitumor effects of CTS-OS, COS and HOS on human HepG2 cells were evaluated *in vitro* cytotoxicity experiments with a wide range of concentrations from 0.75 to 50 μg·mL^−1^. The results showed that CTS-OS has the strongest effect against HepG2 cells with CC_50_ values lower than 25 μg·mL^−1^ (Figure 6). Treatment of a series of CTS-OS COS and HOS on HepG2 cells resulted in apparent toxicity compared with the other cell lines, A549 and PC3 cells (Figures 4 and 5). These results suggest that chitosan oligosaccharides have higher specificity toward human HepG2 cells, while there was slightly increased (<15%) relative cell viability to both cells A549 and PC3 cells. On the other hand, treatment of COS could inhibit tumor growth similar to the relative cell viability of all three cells exposed to CTS-OS at the same concentration. As expected, HOS showed much less tumor cell growth inhibition with CC_50_ values higher than 50 μg·mL^−1^. However, compared to HMWC, HOS showed similar antitumor activity.

The functional properties of chitosan and its depolymerized compounds are mainly dependent upon their solubility, molecular weight and DDA in aqueous media. To overcome these problems, preparation of chitosan oligosaccharides in an active form is obviously gaining importance in many biomedical applications. Although several methods are available for the preparation of oligomeric compounds, development of new effective methods for producing biologically active molecules from chitosan is a major challenge in carbohydrate chemistry to provide sufficient amounts of products needed for fundamental research and their potential application in various fields. Therefore, the main objective of the present study was to evaluate the structural characteristics such as molecular weight and DDA of COS and HOS obtained from the enzymatic hydrolysis of HMWC, and their antitumor activity toward human tumor cells was tested in this study. The results clearly demonstrated that the molecular weight and DDA of chitosan oligosaccharides are important factors for their antitumor activities (Table 3). The relatively smaller size (molecular weight), higher solubility, and the lower degree of deacetylation (DDA) of CTS-OS and COS than HMWC would be promising factors for the development of potential pharmaceuticals or neutraceuticals using these chitosan derivatives.

## 3. Experimental Section

### 3.1. Materials

A high molecular weight of chitosan (HMWC) with approximately 1,900 kDa, 98.5% degree of deacetylation (DDA) was kindly provided by Prof. R. D. Park, Cheonnam University, Korea. A recombinant chitosanase (OHK) was purchased from Kyowa Chemical Ltd. (Japan). All other reagents were used without further purification and were of the highest grade available.

### 3.2. Tumor Cell-Lines

Human PC3 (prostate cancer cell, ATCC No. CRL-1435^TM^), A549 (carcinomic human alveolar basal epithelial cell, ATCC No. CCL-185^TM^) and HepG2 (hepatocellular carcinoma cell, ATCC No. HB-8065^TM^) cells are used as the first choice for evaluating the antitumor effects of chitosan and its oligosaccharides *in vitro*. Cells were grown and maintained in Roswell Park Memorial Institute medium (RPMI) 1640 supplemented with 10% (v/v) heat-inactivated fetal bovine serum (FBS) and 1% penicillin-streptomycin (GIBCO, USA). The cells were maintained at 37 °C under 5% CO_2_.

### 3.3. Preparation of Chitosan-Oligosaccharides

The high molecular weight chitosan (HMWC) was fully dissolved in 1% acetic acid to be 1% (w/v) at room temperature and the pH of the solution was adjusted to pH 5.0 with NaHCO_3_. The chitosan solution was centrifuged at 14,000 rpm for 30 min to remove insoluble materials. Then chitosan was hydrolyzed at 30 °C for 2 h using 0.5 U (nmol reducing sugars/μg/min) of recombinant chitosanase OHK purchased from Kyowa Chemical Ltd., and the reactant was boiled to quench the reaction at 100 °C for 10 min. Following, an ultra-filtration membrane filter with molecular weight cut off (MWCO) of 10 kDa was used to remove denatured protein, insoluble materials and separate water-soluble chitosan-oligosaccharides CTS-OS. Obtained CTS-OS was further free-dried, and kept at 4 °C until use. For cytotoxicity test against tumor cells, solutions (1% acetic acid) of HMWC and its enzyme hydrolyzed products (CTS-OS, HOS, and COS) were adjusted to pH 5.0 with 1 M NaHCO_3_ and then applied to each tumor cell culture.

### 3.4. Gel-filtration Chromatography

CTS-OS obtained after digestion of HMWC was further fractionated using a gel-filtration column chromatograph (Φ1.0 cm × L 30 cm) packed with Bio Gel-P 4 (Bio-RAD, USA) gel, which had been pre-equilibrated with a volatile buffer consisting of ammonia/formic acid (pH 7.5). A total of 500 mg CTS-OS dissolved in 1.0 mL of the same buffer was applied to a gel-filtration column and eluted at a flow-rate of 5 μL per min until no carbohydrates were detected using the Nelson-Somogyi method [17].

### 3.5. Determination of Molecular Weight and Deacetylation Degree (DDA) of CTS-OS by MALDI-TOF MS Analysis

The two major carbohydrate-positive fractions obtained were applied to matrix-assisted laser desorption/ionization-time of flight mass spectrometry (MALDI-TOF MS) analysis. The molecular weight and deacetylation degree (DDA) of CTS-OS obtained from the enzymatic hydrolysis of chitosan was determined by matrix-assisted laser desorption/ionization-mass spectrometry (MALDI-TOF MS) analysis (Voyager-DE TM STR Biospectrometry Workstation, Applied Biosystems Inc., NCIRF, Korea). The molecular weight and DDA of CTS-OS was calculated based on the molecular weight of glucosamine (GlcN) and *N*-acetylglucosamine (GlcNAc).

### 3.6. Cytotoxicity against Tumor Cells *in Vitro*

The viability of tumor cell lines PC3, A549, and HepG2 human-derived against HMWC and its oligosaccharides was determined by the 3-(4,5-dimethylthiazol-2-yl)-2,5-diphenyl tetrazolium bromide (MTT, Sigma) reduction assay [18]. Tumor cells were placed in RPMI 1640 supplemented with 10% FBS (PAA) and 1% penicillin-streptomycin (GIBCO) at 1 × 10^4^ cells/well in 24-well culture plates. After, the cells were cultured overnight at 30 °C, and the medium was changed to fresh RPMI 1640. Then cells were exposed to the indicated amounts of chitosan and its oligosaccharides for 24 h. After monolayer cultivation for 24 h, the medium was removed and 100 μL of the maintenance medium (MM) and different concentrations of samples were added to each well and the samples were then incubated for an additional 24 h. After 24 h incubation, 20 μL of MTT ((3,4,5-dimetylthiazol-2-yl)-2,5- diphenyl tetrazonium bromide)) solution (5 mg·mL^−1^) was added to each well of the plate and re-incubated for 4 h. After removal of the supernatant, 100 μL of DMSO were added to each well to dissolve the crystals completely and the absorbance was measured at 570 nm using an ELISA Reader (Bio-Rad, USA). The cytotoxicity was expressed as the 50% cytotoxic concentration (CC_50_), the concentration of samples needed to inhibit the cell growth by 50%.

### 3.7. Statistical Analysis

All data were expressed as the means ± standard Deviation (SD), which are representative of at least three different experiments. Comparison between individual data points of each experiment were conducted using Student’s *t*-test. All *p*-values of <0.1 were considered to be significant.

## 4. Conclusions

In the present study, we report antitumor effects of HMWC, CTS-OS, COS and HOS toward three different human tumor cell lines used as a model for the experiments *in vitro*. Our results show that, among other compounds, CTS-OS and COS obtained from enzymatic hydrolysis of chitosan and identified by MALDI-TOF MS analysis showed significant antitumor activities against human A549, PC3, and HepG2 tumor cells. More importantly, no significant antitumor effect of HMWC and HOS was observed. Taken together, these results strongly suggest that properties of chitosan, such as the average molecular weight and the degree of *N*-deacetylation (DDA), might be important factors for the exhibition of antitumor activity *in vitro*. Since CTS-OS and COS have higher solubility in water and narrow molecular weight distribution ensuring reproducible pharmacological properties compared to HOS and HMWC, they may provide valuable information for the further development of antitumor agents deriving from chitosan.

## Figures and Tables

**Table 1:** Preparation of chitosan oligosaccharides.

Chitosan oligosaccharides	Yields(%)*	DDA (%)**
Dimer	18.8	100
Trimer	24.8	100
Tetramer	24.9	100
Pentamer	17.7	100
Hexamer	7.1	833.3
Heptamer	3.3	85.7
Octamer	3.4	87.5

* The production yields of each oligosaccharide obtained by enzymatic hydrolysis of chitosan were calculated based on MALDI-TOF MS analysis data.

** DDA, degree of deacetylation of chitosan oligosaccharides.

**Table 2:** Prediction of the molecular weight of chitosan oligosaccharides (CTS-OS) by MALDI-TOF MS analysis.

Chitosan oligosaccharides (CTS-OS)	*m/z*
(GlcN)_n_, n = 2–5	360, 524, 685, 846
(GlcN)_5_ + GlcNAc	1049
(GlcN)_5_ + GlcNAc + GlcN Or (GlcN)_6_ + GlcNAc	1210
(GlcN)_5_ + GlcNAc + (GlcN)_2_ Or (GlcN)_7_ + GlcNAc	1371
(GlcN)_5_ + GlcNAc + (GlcN)_2_ + GlcNAc or (GlcN)_7_ + GlcNAc	1574
(GlcN)_5_ + GlcNAc + (GlcN)_2_ + GlcNAc + GlcN Or (GlcN)_8_ + (GlcNAc)_2_	1735
(GlcN)_5_ + GlcNAc + (GlcN)_2_ + GlcNAc + (GlcN)_2_ Or (GlcN)_9_ + (GlcNAc)_2_	1896
(GlcN)_5_ + GlcNAc + (GlcN)_2_ + GlcNAc + (GlcN)_2_ + GlcNAc Or (GlcN)_9_ + (GlcNAc)_3_	2099
(GlcN)_5_ + GlcNAc + (GlcN)_2_ + GlcNAc + (GlcN)_2_ + GlcNAc + GlcN Or (GlcN)_10_ + (GlcNAc)_3_	2260
GlcN)_5_ + GlcNAc + (GlcN)_2_ + GlcNAc + (GlcN)_2_ + GlcNAc + (GlcN)_2_ Or (GlcN)_11_ + (GlcNAc)_3_	2421
GlcN)_5_ + GlcNAc + (GlcN)_2_ + GlcNAc + (GlcN)_2_ + GlcNAc + (GlcN)_2_ + GlcNAc Or (GlcN)_11_ + (GlcNAc)_4_	2625

**Table 3:** Effect of the degree of deacetylation (DDA) of chitosan derivatives on antitumor activity.

Samples	DDA (%)	CC_50_ (μg·mL^−1^)*		
		PC3	A549	HepG2
HMWC	98.5	50	50	50
CTS-OS	98.5	25	25	25
COS	100	25	5	12.5
HOS	85.5	50	50	50

* CC_50_ (μg·mL^−1^) the concentration of each sample required for 50% cell death.
